# Developing a nomogram based on admission AGR and CLR to predict subsequent surgical intervention in pediatric acute hematogenous osteomyelitis

**DOI:** 10.1038/s41598-026-57260-4

**Published:** 2026-06-08

**Authors:** Chaochen Zhao, Yuhan Sun, Zhiye Guan, Chenghui Ke, Xiaodong Wang, Ziming Zhang

**Affiliations:** 1 Department of Orthopedics, Shanghai Children’s Hospital, School of Medicine, Shanghai Jiao Tong University, Shanghai, China; 2 Department of Orthopedics, Children’s Hospital of Soochow University, Suzhou, Jiangsu Province China

**Keywords:** Acute hematogenous osteomyelitis, Pediatrics, Surgical intervention, Nomogram, Albumin-to-globulin ratio, C-reactive protein-to-lymphocyte ratio, Biomarkers, Diseases, Medical research, Risk factors

## Abstract

Pediatric acute hematogenous osteomyelitis (AHO) can progress rapidly, but admission findings associated with subsequent operative management remain incompletely defined. In this two-center retrospective study, we evaluated the albumin-to-globulin ratio (AGR) and C-reactive protein-to-lymphocyte ratio (CLR) as admission biomarkers associated with surgical intervention during the index hospitalization. Because this outcome is partly clinician-determined, the model was designed to estimate subsequent surgical management rather than the biological necessity for surgery. We included 120 children admitted between 2015 and 2024 (conservative treatment, n = 58; surgical treatment, n = 62). Clinical characteristics, imaging findings, microbiological results, and laboratory variables measured within 2 h of admission and before antibiotic initiation were reviewed. AGR and CLR were modeled as continuous predictors in Firth’s penalized logistic regression and used to construct an admission-based nomogram. Lower AGR and higher CLR were independently associated with surgical intervention (AGR: OR 0.056, 95% CI 0.012–0.220, *P* < 0.001; CLR: OR 1.008, 95% CI 1.002–1.018, *P* = 0.008). The model showed moderate discrimination (AUC = 0.755; optimism-corrected AUC = 0.749) and satisfactory calibration within this cohort (Hosmer–Lemeshow *P* = 0.922; Brier score = 0.198), with internal validation performed using stratified tenfold cross-validation and 1000 bootstrap resamples. At the Youden-derived cutoff of 0.552, sensitivity and specificity were 0.645 and 0.759, respectively. Decision curve analysis suggested potential net benefit mainly across threshold probabilities of approximately 10–60%. These findings indicate that admission AGR and CLR are associated with later clinician-determined surgical intervention and may help identify children who require closer observation, earlier imaging prioritization, and timely reassessment. The nomogram should be considered exploratory and hypothesis-generating; independent external validation is required before routine clinical use.

## Introduction

Acute hematogenous osteomyelitis (AHO) in children is a rapidly progressing bone and joint infection caused by the hematogenous dissemination of pathogens. Its annual incidence ranges from 2 to 20 cases per 100,000 children in high-income countries, with a markedly higher incidence of 43 to 200 per 100,000 children reported in lower-income regions^1–4^. Clinically, AHO typically manifests as fever, localized pain, swelling, and restricted limb function. Delays in diagnosis or initiation of treatment facilitate rapid intraosseous spread of infection, resulting in tissue necrosis, abscess formation, and potential joint involvement^5^. These complications significantly heighten the risk of growth plate damage, subsequent growth disturbances, skeletal deformities, chronic osteomyelitis, and life-threatening sepsis^6^.

Staphylococcus aureus remains the predominant pathogen, accounting for 70–90% of pediatric AHO cases^5,7^. The prevalence of methicillin-resistant Staphylococcus aureus (MRSA) has increased in many settings^8^. Compared with methicillin-susceptible Staphylococcus aureus (MSSA), MRSA infections are often accompanied by a more intense inflammatory response, higher abscess rates, longer antibiotic courses, and a greater likelihood of surgical intervention, which complicates management^9,10^. Empirical intravenous antibiotics remain the mainstay of treatment, but surgical debridement or drainage is often required when imaging shows intramedullary abscesses or joint involvement^11^. Delayed surgical intervention may increase acute-phase complications, especially when abscess formation is already present^5^. However, explicit guidance on surgical indications and timing remains limited. In practice, surgery is commonly considered when disease progresses rapidly, when clinical improvement is insufficient after 48–96 h of antibiotics, or when magnetic resonance imaging (MRI) shows intramedullary abscess formation or periosteal elevation exceeding 2 mm^11,12^. Timely MRI may be difficult in primary care settings, and young children may require sedation. A simple, rapid, admission-based tool that supports early risk stratification for subsequent surgical management may therefore be useful, particularly when used alongside serial clinical assessment and timely imaging evaluation.

Previous research by Guo et al. identified elevated neutrophil counts at admission and MRSA infection as potential predictors for surgical intervention, offering valuable preliminary evidence within this domain^12^. However, the prevalence of MRSA varies considerably by region, and conventional bacterial cultures require 48–72 h for results, highlighting the critical need for more rapidly available and universally applicable biomarkers^8^.

The albumin to globulin ratio (AGR) and C-reactive protein to lymphocyte ratio (CLR) are simple, routinely available inflammatory biomarkers that have attracted increasing attention because of their potential prognostic value across a wide range of diseases. Xiao et al. reported that CLR is an important predictor of short-term adverse clinical outcomes in patients with SARS-CoV-2 BA.2.2 infection^13^. Balta et al. suggested that CLR may also serve as a useful screening tool for the diagnosis of periprosthetic joint infection^14^. In the context of periprosthetic joint infection, several studies have indicated that globulin and AGR are promising serum biomarkers that can improve diagnostic accuracy^15^. For example, Shang et al. showed that serum globulin and AGR have good diagnostic performance for periprosthetic joint infection and can accurately predict culture results and persistent infection, thereby guiding the optimal timing of second-stage reimplantation^16^. In addition, a meta-analysis by Chen et al. suggested that AGR is a reliable serum biomarker for detecting hip or knee PJI^17^. However, evidence regarding the usefulness of AGR and CLR in predicting subsequent surgical intervention in children with AHO remains scarce.

The present study therefore evaluated whether admission AGR and CLR could estimate the probability of surgical intervention during the index hospitalization in children with AHO and support construction of an admission-based nomogram. We framed the endpoint as clinician-determined surgical intervention rather than an objective measure of disease severity or biological necessity for surgery. The model was intended to complement serial clinical assessment, microbiological evaluation, and imaging-based decision-making.

## Materials and methods

This retrospective study was approved by the Ethics Committee of Shanghai Children’s Hospital and the Ethics Committee of Children’s Hospital of Soochow University (approval numbers: 2021KS024 and 2025R044E01). The requirement for written informed consent was waived by the Ethics Committee of Shanghai Children’s Hospital and the Ethics Committee of Children’s Hospital of Soochow University because of the retrospective nature of the study and the use of anonymized clinical data. All methods were performed in accordance with the relevant guidelines and regulations and with the principles of the Declaration of Helsinki. The adequacy of the sample size was supported by methodological considerations: the final prediction model included two predictors and 62 surgical events among 120 patients, yielding an events-per-variable ratio of 31, which exceeds the commonly recommended threshold of 10–20 events per variable for logistic regression. Therefore, the available sample size was considered adequate for developing a parsimonious two-predictor model, although the modest cohort size still limits precision and generalizability.

### Study design and participants

Patients hospitalized with acute hematogenous osteomyelitis between January 2015 and December 2024 in the two hospitals were enrolled. The inclusion criteria were as follows: (1) confirmed diagnosis of AHO; (2) symptom duration ≤ 14 days prior to admission without prior antibiotic exposure before baseline laboratory evaluation; and (3) age between 1 month and 18 years. Exclusion criteria included: (1) underlying hematologic, rheumatologic, cardiovascular, malignant, or chronic systemic diseases; (2) secondary infections following open fractures or surgical interventions; and (3) incomplete medical records. AHO was diagnosed based on positive cultures obtained from blood, purulent material, synovial fluid, or infected bone tissue. For culture-negative patients, presumptive AHO diagnosis required compatible clinical symptoms, supportive laboratory and imaging evidence, and clear clinical improvement within 48 h following the initiation of antibiotic therapy^11,18^.

### Standard treatment protocol

Empirical intravenous antibiotic therapy, typically cefuroxime or ceftriaxone, was initiated on admission. Antibiotic regimens were then adjusted according to microbiological culture and susceptibility results. Patients who improved clinically, with recovery of gastrointestinal function, declining inflammatory biomarkers, and two consecutive C-reactive protein (CRP) levels below 8 mg/L, were transitioned to oral antibiotic therapy. Patients who required surgery or had substantial abscess formation continued intravenous antibiotics until clinical stability was achieved, followed by oral treatment. Surgical procedures included bone debridement, abscess drainage, and arthrotomy. In both participating centers, pediatric bone and joint infections were managed primarily within pediatric orthopedic departments. Pediatric internal medicine or infectious disease physicians provided antimicrobial guidance when needed, whereas operative indications and timing were determined mainly by pediatric orthopedic surgeons. Surgical decision-making followed guideline-concordant and literature-supported criteria, including persistent symptoms despite 48–96 h of antibiotics, ongoing fever or local inflammation, abscess formation, concomitant septic arthritis, or MRI-documented periosteal elevation exceeding 2 mm^11,19^. This specialty-based workflow and the use of broadly shared criteria may have limited nonsystematic inter-physician variation and may improve the reproducibility of the outcome in future validation studies. Still, because decisions were made in routine care rather than under a prospective adjudication protocol, residual variation in physician judgment, MRI availability and timing, interpretation of abscess burden, and center-specific practice patterns could not be fully eliminated.

### Data collection

Data were extracted from electronic medical records with a standardized case report form. Demographic and clinical variables included sex, age, infection site, admission temperature, concomitant suppurative arthritis, symptom duration before admission, surgical intervention status, and microbiological culture results. Laboratory tests performed within 2 h of admission included erythrocyte sedimentation rate (ESR), white blood cell (WBC) count, neutrophil count, lymphocyte count, CRP, serum albumin, and serum globulin. In both centers, routine hematological and biochemical measurements were performed by institutional clinical laboratories under local quality-control procedures, and values were reviewed in their original units before harmonization for analysis. AGR was calculated as serum albumin (g/L) divided by serum globulin (g/L), and CLR as CRP (mg/L) divided by lymphocyte count (× 10⁹/L). Fever at admission was defined as a body temperature > 38.5 °C, consistent with the threshold used in the Kocher algorithm for children. All baseline blood samples were collected before antibiotic therapy was started. Patients were assigned to the conservative or surgical group according to management during the index hospitalization. This outcome therefore reflected not only baseline disease severity, but also subsequent clinical evolution, imaging findings, microbiological information, physician judgment, and center-level practice.

### Statistical analysis

All statistical analyses were performed using IBM SPSS Statistics version 26.0 (IBM Corporation, Armonk, NY, USA) and R software version 4.5.1 (R Foundation for Statistical Computing, Vienna, Austria). Continuous variables with normal distributions were reported as mean ± standard deviation, and non-normally distributed variables as median (P25, P75). Categorical variables were reported as frequencies and percentages. Missingness was assessed at the variable level. Because missing data were sparse (< 5% for all variables) and appeared sporadic on chart review, median imputation was used in the primary analysis to preserve sample size and avoid instability from more complex imputation models in a modest cohort. Median imputation may underestimate variability; therefore, the primary model was also re-estimated in a complete-case sensitivity analysis after excluding observations with missing predictor data. Multiple imputation was not used as the primary approach because the very low fraction of missing data was expected to add little information while introducing additional modeling assumptions. Between-group comparisons used the independent-samples t-test for normally distributed variables, the Mann–Whitney U test for non-normally distributed variables, and the χ^2^ test for categorical variables.

The primary outcome was surgical intervention during the index hospitalization (yes/no). We treated this as a decision-level endpoint: it captured treatment delivered in routine care, not an adjudicated biological need for surgery. Because operative decisions were shaped by baseline disease severity, subsequent clinical progression, imaging findings, microbiological information, and physician judgment, the model was intended to estimate the likelihood of subsequent clinician-determined surgical management within the participating centers, rather than to serve as a stand-alone measure of biological necessity for surgery. Univariable logistic regression analyses first explored associations between candidate predictors and surgical intervention. Given the number of events (n = 62), the primary multivariable model was intentionally kept parsimonious and admission-based. AGR and CLR were prespecified as core predictors because they are biologically plausible, supported by prior evidence, and available at admission. Data-driven selection procedures, including stepwise regression, were avoided. Variables that were unavailable at the intended point of use, reflected downstream decision-making, or were mathematically coupled with composite biomarkers were not entered into the primary model. Thus, microbiologically confirmed MRSA infection was excluded because culture results are typically unavailable at admission; detailed MRI features such as abscess size were not incorporated because imaging was not uniformly available at baseline; CRP was not entered with CLR because CLR includes CRP; and albumin or globulin were not entered with AGR. These choices were consistent with the intended early-use setting of the model, but they may introduce residual confounding, particularly because MRSA infection, abscess burden, and septic arthritis can influence operative decisions. Potential nonlinear associations between the continuous predictors and the outcome were examined with restricted cubic splines before multivariable modeling. Spline terms were retained when the test for nonlinearity yielded *P* < 0.05; otherwise, predictors were modeled as linear terms. Multicollinearity was assessed using variance inflation factors, with VIF < 5 indicating no substantial collinearity. Multivariable analysis used Firth’s penalized likelihood logistic regression to reduce small-sample bias. A prespecified sensitivity analysis fitted an expanded Firth model that added fever at admission, symptom duration, and neutrophil count to AGR and CLR. Individual predicted probabilities were calculated from the model coefficients and intercept, and a nomogram was constructed using the rms package.

Model discrimination was evaluated using the area under the receiver operating characteristic curve (AUC) with 95% confidence intervals. Calibration was assessed with the Hosmer–Lemeshow goodness-of-fit test, calibration plots, and the Brier score. Internal validation used 1,000 bootstrap resamples and stratified tenfold cross-validation. The apparent AUC was calculated from the final model fitted to the full dataset, whereas the cross-validated AUC was reported separately as the mean of fold-specific out-of-fold AUC values. Because no external or temporal validation cohort was available, these procedures were used to assess optimism and internal stability, not transportability. Decision curve analysis evaluated the model’s net clinical benefit across threshold probabilities. We interpreted the decision curve as evidence for model-guided triage rather than direct treatment selection, recognizing that misclassification has asymmetric consequences: false-negative predictions may delay surgical reassessment, whereas false-positive predictions may lead to unnecessary imaging, consultation, or intervention. A *p*-value < 0.05 was considered statistically significant.

## Results

**Figure 1:**
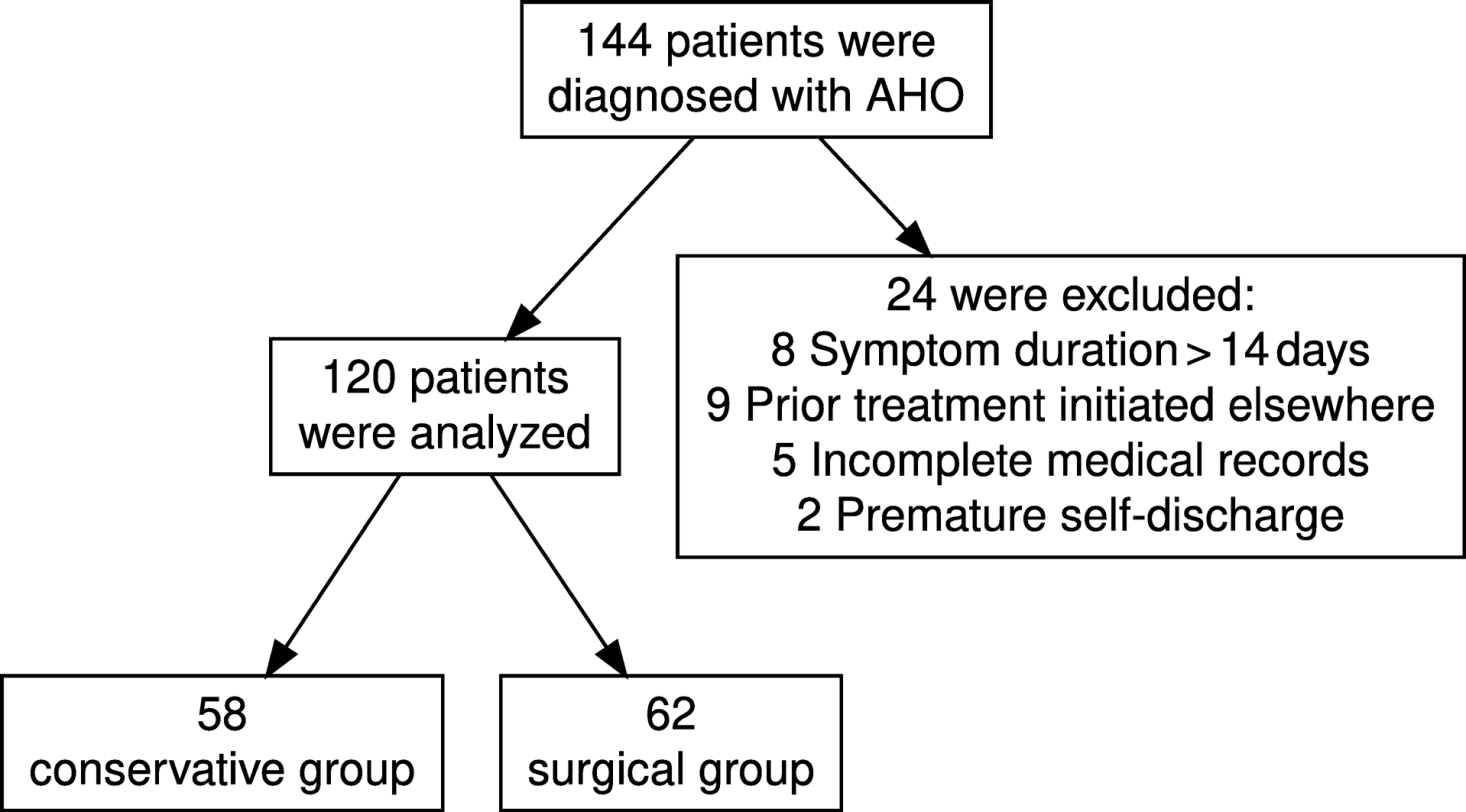
The flow chart of the study.

In total, 144 patients were diagnosed with AHO. Of these, 24 were excluded from the study for the following reasons: 8 cases presented with symptom duration exceeding 14 days, and 16 had issues related to prior care or documentation. The latter group included 9 patients who had already begun treatment at other institutions, 5 with incomplete medical records, and 2 who left the hospital prematurely. Consequently, the final analysis cohort comprised 120 pediatric AHO cases (Fig. 1).

### General information

Of the 120 patients, 76 (63.3%) were male and 44 (36.7%) female, with a median age of 8 years (IQR, 3.2–10.4). The most frequently affected anatomical sites were long bone metaphyses, primarily involving the femur (35 cases, 29.3%), tibia (n = 29, 24.3%), humerus (14 cases, 11.7%), and calcaneus (12 cases, 10.0%). Less commonly involved sites included the fibula (11 cases, 9.3%), pelvis/ilium (5 cases, 4.3%), phalanges (5 cases, 4.3%), radius ( 2 cases, 1.8%), ulna (2 cases, 1.8%), and singular cases each involving the metatarsal, clavicle, patella, and navicular bones (each 1 case, 0.8%). Suppurative arthritis coexisted in 21 patients (17.5%).

Microbiological cultures were performed in 113 cases. Blood cultures yielded positive results in 22 of 101 tested patients (21.8%), whereas abscess or bone tissue cultures were positive in 49 of 61 patients (80.3%). Among all pathogens identified, Staphylococcus aureus predominated (56/60, 93.4%), including 40 MSSA and 16 MRSA. Non-staphylococcal isolates included *Pseudomonas aeruginosa* (*n* = 2), *Salmonella spp.* (*n* = 1), and *Klebsiella oxytoca* (*n* = 1). MRI was obtained in 105 patients (87.5%), identifying intramedullary abscess formation in 49 cases (40.8%). Patients were classified into conservative (*n* = 58) and surgical treatment (*n* = 62) groups based on therapeutic approaches.

### Univariate comparisons between conservative and surgical groups

Chi-square and non-parametric tests showed no significant differences in age, sex, presence of fever at admission, duration from symptom onset to admission, or lymphocyte count between the conservative and surgical groups (all *P* > 0.05). However, MRSA infection and concomitant suppurative arthritis were significantly more frequent in the surgical group than in the conservative group (*P* = 0.045 and *P* = 0.046, respectively). In addition, patients in the surgical group had significantly higher WBC counts, ESR, CRP levels, neutrophil counts, serum globulin levels, and CLR values, whereas serum albumin levels and AGR were significantly lower compared with those in the conservative group (all *P* < 0.05) (Table 1).

**Table 1:** Univariate comparisons between conservative and surgical groups.

Variables	Total cases (*n* = 120)	Conservative (*n* = 58)	Surgery (*n* = 62)	*P*
Age, years, median(IQR)	8.0 (3.2, 10.4)	7.3 (1.6, 11.1)	8.1 (4.5, 9.9)	0.603
Sex,* n* (%)				0.781
Male	76 (63.3)	36 (62.1)	40 (64.5)	–
Female	44 (36.7)	22 (37.9)	22 (35.5)	–
Fever at admission,* n* (%)	54 (45.0)	27 (46.6)	27 (43.5%)	0.741
MRSA,* n* (%)	16 (13.3)	4 (6.9)	12 (19.4%)	0.045
Suppurative arthritis,* n* (%)	21 (17.5)	6 (10.3)	15 (24.2%)	0.046
Symptom duration before admission, days	6.0 (3.3, 7.0)	5.0 (3, 7)	6.0 (3.8, 8.0)	0.098
WBC count, × 10^9^/L, median (IQR)	11.9 (9.0, 15.4)	10.6 (8.3, 14.5)	12.9 (9.5, 16.0)	0.039
ESR on admission, mm/h, median (IQR)	46.5 (24.3, 68.8)	42.0 (20.8, 65.0)	50.5 (33.8, 80.1)	0.038
CRP on admission, mg/L, median (IQR)	50.0 (21.9, 94.5)	41.2 (12.5, 74.19)	52.1 (29.4, 112.9)	0.022
Neutrophil count, × 10^9^/L, median (IQR)	7.6 (4.8, 10.1)	6.4 (3.8, 9.3)	8.4 (6.8, 11.4)	0.003
Lymphocyte count, × 10^9^/L, median (IQR)	2.5 (1.6, 3.8)	2.7 (1.9, 4.1)	2.5 (1.4, 3.7)	0.149
Albumin on admission, g/L, median (IQR)	41.9 (38.5, 44.4)	43.0 (40.5, 44.5)	40.0 (36.8, 42.8)	0.001
Globulin on admission, g/L, median (IQR)	28.6 (25.0, 31.9)	27.6 (23.5, 30.5)	29.9 (26.9, 33.4)	0.005
AGR, median (IQR)	1.4 (1.3, 1.7)	1.6 (1.4, 1.9)	1.4 (1.2, 1.5)	< 0.001
CLR, median (IQR)	17.1 (6.8, 49.0)	13.8 (3.9, 35.3)	21.2 (9.0, 64.1)	0.019

*MRSA* methicillin-resistant Staphylococcus aureus, *WBC* white blood cell count, *ESR* Erythrocyte sedimentation rate, *CRP* C-reactive protein, *AGR* Albumin to globulin ratio, *CLR* C-reactive protein to lymphocyte ratio.

### Univariable logistic regression analysis

Univariable logistic regression analysis demonstrated that higher ESR (OR 1.015, 95% CI 1.003–1.028, *P* = 0.019), higher CRP level (OR 1.008, 95% CI 1.001–1.016, *P* = 0.019), higher neutrophil count (OR 1.102, 95% CI 1.009–1.203, *P* = 0.030), higher serum globulin level (OR 1.112, 95% CI 1.034–1.196, *P* = 0.004), and higher CLR (OR 1.011, 95% CI 1.002–1.020, *P* = 0.022) were significantly associated with increased odds of surgical intervention. In contrast, serum albumin (OR 0.866, 95% CI 0.788–0.952, *P* = 0.003) and AGR (OR 0.054, 95% CI 0.013–0.229, *P* < 0.001) were inversely associated with surgical intervention. MRSA infection (OR 3.240, 95% CI 0.981–10.706, *P* = 0.054) and concomitant suppurative arthritis (OR 2.766, 95% CI 0.992–7.714, *P* = 0.052) showed borderline associations with surgical intervention, whereas WBC count was not significantly associated with the outcome (OR 0.994, 95% CI 0.969–1.020, *P* = 0.658) (Table 2).

**Table 2:** Univariable logistic regression analyses of factors associated with surgery.

Variables	Β	Standard Error	*X*^*2*^	P	Odds Ratio	95% CI for Odds Ratio	
						Lower	Upper
MRSA	1.176	0.610	3.717	0.054	3.240	0.981	10.706
Suppurative arthritis	1.017	0.523	3.779	0.052	2.766	0.992	7.714
WBC	− 0.006	0.013	0.196	0.658	0.994	0.969	1.020
ESR	0.015	0.006	5.527	0.019	1.015	1.003	1.028
CRP	0.008	0.004	5.527	0.019	1.008	1.001	1.016
Neutrophil count	0.097	0.045	4.696	0.030	1.102	1.009	1.203
Albumin	− 0.144	0.049	8.797	0.003	0.866	0.787	0.952
Globulin	0.106	0.037	8.225	0.004	1.112	1.034	1.196
AGR	− 2.910	0.731	15.824	< 0.001	0.054	0.013	0.229
CLR	0.011	0.005	5.226	0.022	1.011	1.002	1.020

*MRSA* methicillin-resistant Staphylococcus aureus, *WBC* white blood cell count, *ESR* Erythrocyte sedimentation rate, *CRP* C-reactive protein, *AGR* Albumin to globulin ratio, *CLR* C-reactive protein to lymphocyte ratio.

### Nonlinearity and multicollinearity assessment

Restricted cubic spline analyses were performed to examine potential nonlinear associations between AGR or CLR and the risk of surgical intervention. The nonlinear components were not statistically significant for either variable (AGR: χ^2^ = 2.88, *P* = 0.237; CLR: χ^2^ = 0.68, *P* = 0.712), indicating that both AGR and CLR exhibited approximately linear relationships with the risk of surgical intervention within the observed range. Therefore, linear terms of AGR and CLR were retained in the final multivariable model. Variance inflation factor values for both predictors were close to 1 (AGR = 1.00; CLR = 1.00), suggesting no evidence of substantial multicollinearity among the explanatory variables.

### Multivariate regression analysis and the development of a predictive model

**Figure 2:**
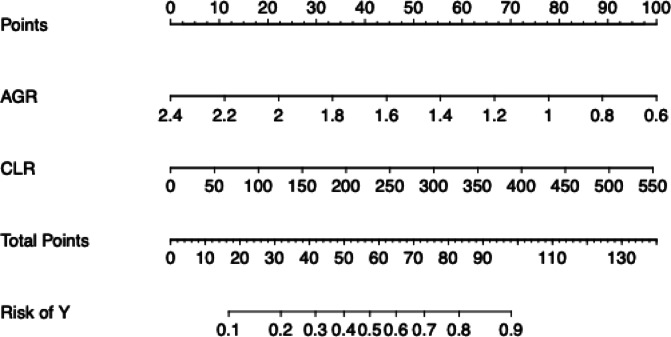
Nomogram for visualizing individualized predicted probabilities of subsequent clinician-determined surgical intervention in pediatric patients with AHO.

In the multivariable Firth’s penalized logistic regression analysis, the overall model was statistically significant (likelihood ratio χ^2^ = 26.47, *P* < 0.001). AGR and CLR were both independently associated with surgical intervention. AGR was inversely associated with the likelihood of subsequent surgical intervention (OR 0.056, 95% CI 0.012–0.220, *P* < 0.001), whereas CLR showed a positive association (OR 1.008, 95% CI 1.002–1.018, *P* = 0.008). AGR and CLR were retained as the core predictors because they were prespecified, available at admission, biologically interpretable, and suitable for a parsimonious early risk-stratification model. The resulting penalized logistic regression equation was: logit [P(surgical intervention = 1)] = 3.969 − 2.885 × AGR + 0.008 × CLR (Table 3). A nomogram was then created to visualize individualized predicted probabilities and support future validation (Fig. 2).

**Table 3:** Firth logistic regression analyses for factors associated with surgery.

Variables	Β	Standard Error	*X*^2^	P	Odds Ratio	95% CI for Odds Ratio	
						Lower	Upper
AGR	− 2.885	0.739	19.273	< 0.001	0.056	0.012	0.220
CLR	0.008	0.0039	6.982	0.008	1.008	1.002	1.018
Intercept	3.969	1.098	15.524	< 0.001	52.912	–	

* AGR* Albumin to globulin ratio, *CLR* C-reactive protein to lymphocyte ratio.

### Sensitivity analysis

In the prespecified sensitivity analysis, an expanded Firth penalized logistic regression model was fitted by adding fever at admission, symptom duration, and neutrophil count. AGR (OR 0.079, 95% CI 0.017–0.367, *P* = 0.001) and CLR (OR 1.010, 95% CI 1.002–1.018, *P* = 0.018) remained independently associated with surgical intervention, whereas fever at admission, symptom duration, and neutrophil count were not statistically significant (Table 4). The expanded model had an AUC of 0.777, a modest increase compared with the primary model (Table 5). In the complete-case sensitivity analysis used to assess median imputation, the direction and magnitude of the AGR and CLR associations were materially unchanged. These findings support the stability of the two-biomarker model within this cohort, although residual confounding by non-admission variables such as microbiological results, abscess characteristics, or concomitant septic arthritis remains possible.

**Table 4:** Sensitivity analysis: expanded Firth penalized logistic regression model for factors associated with surgery.

Variables	Β	Standard Error	*X*^2^	P	Odds Ratio	95% CI for Odds Ratio	
						Lower	Upper
AGR	− 2.538	0.784	10.476	0.001	0.079	0.017	0.367
CLR	0.01	0.004	5.549	0.018	1.01	1.002	1.018
Fever at admission	− 0.148	0.438	0.115	0.735	0.862	0.365	2.036
Symptom duration before admission	0.103	0.07	2.196	0.138	1.109	0.967	1.271
Neutrophil count	0.006	0.052	0.012	0.914	1.006	0.908	1.114
Intercept	2.814	1.486	3.586	0.058	16.676	–	

*AGR* Albumin to globulin ratio, *CLR* C-reactive protein to lymphocyte ratio.

**Table 5:** Receiver operating characteristic curves analysis for predicting surgery.

Variables	AUC	Cutoff value	Sensitivity	Specificity	PPV	NPV	Youden index	Accuracy
AGR	0.716	1.61	0.839	0.483	0.634	0.737	0.321	0.667
CLR	0.624	49.44	0.371	0.879	0.767	0.567	0.250	0.617
Nomogram	0.755	0.552	0.645	0.759	0.741	0.667	0.404	0.700
Expanded sensitivity model	0.777	0.551	0.645	0.793	0.769	0.676	0.438	0.717

*AUC* area under the curve, *PPV* indicates positive predictive value, *NPV* negative predictive value, *AGR* Albumin to globulin ratio, *CLR* C-reactive protein to lymphocyte ratio.

### Validation of the nomogram

**Figure 3:**
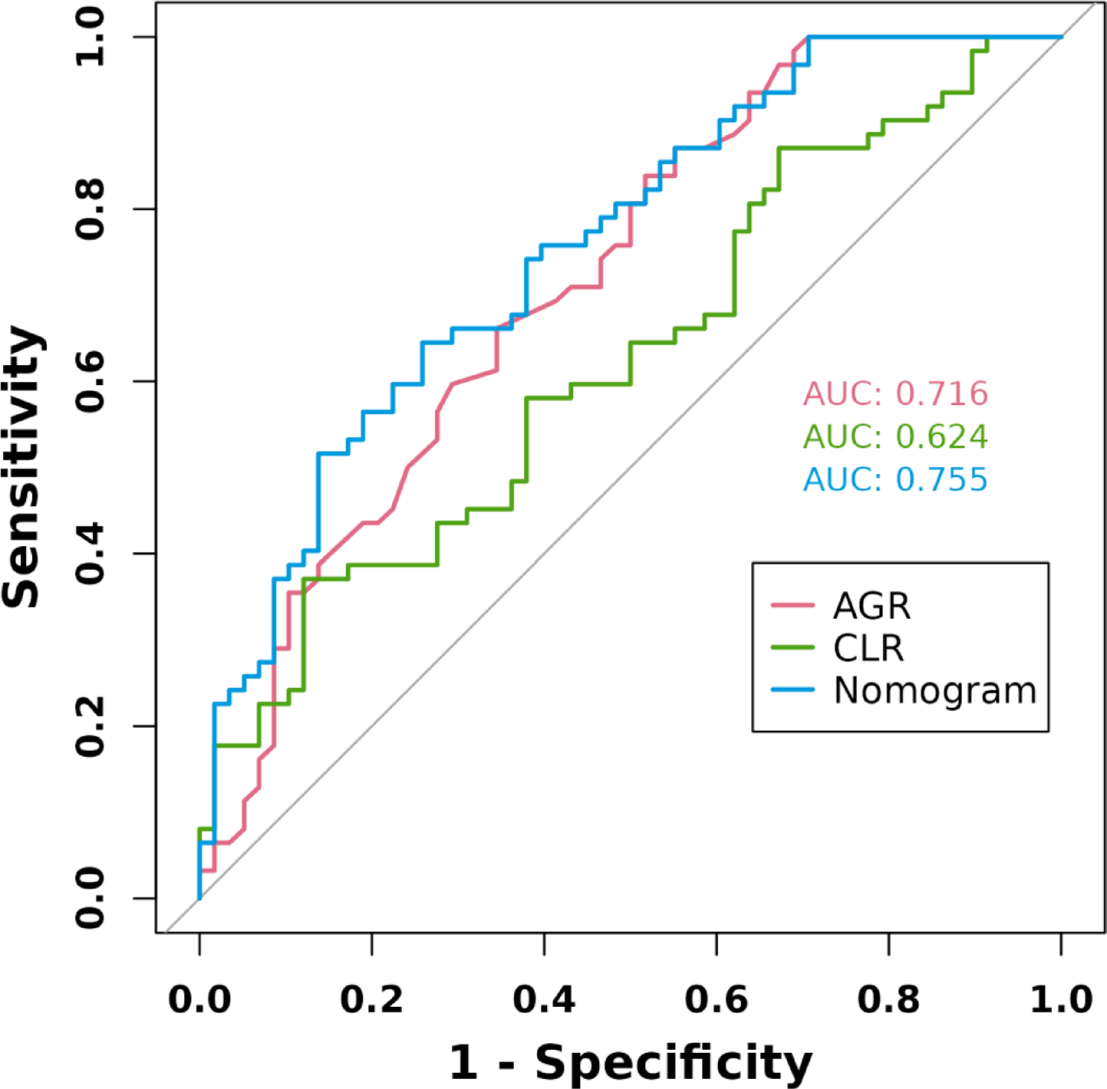
ROC curve for predicting subsequent clinician-determined surgical intervention in AHO patients. ROC, receiver operating characteristic; AUC, area under the curve.

**Figure 4:**
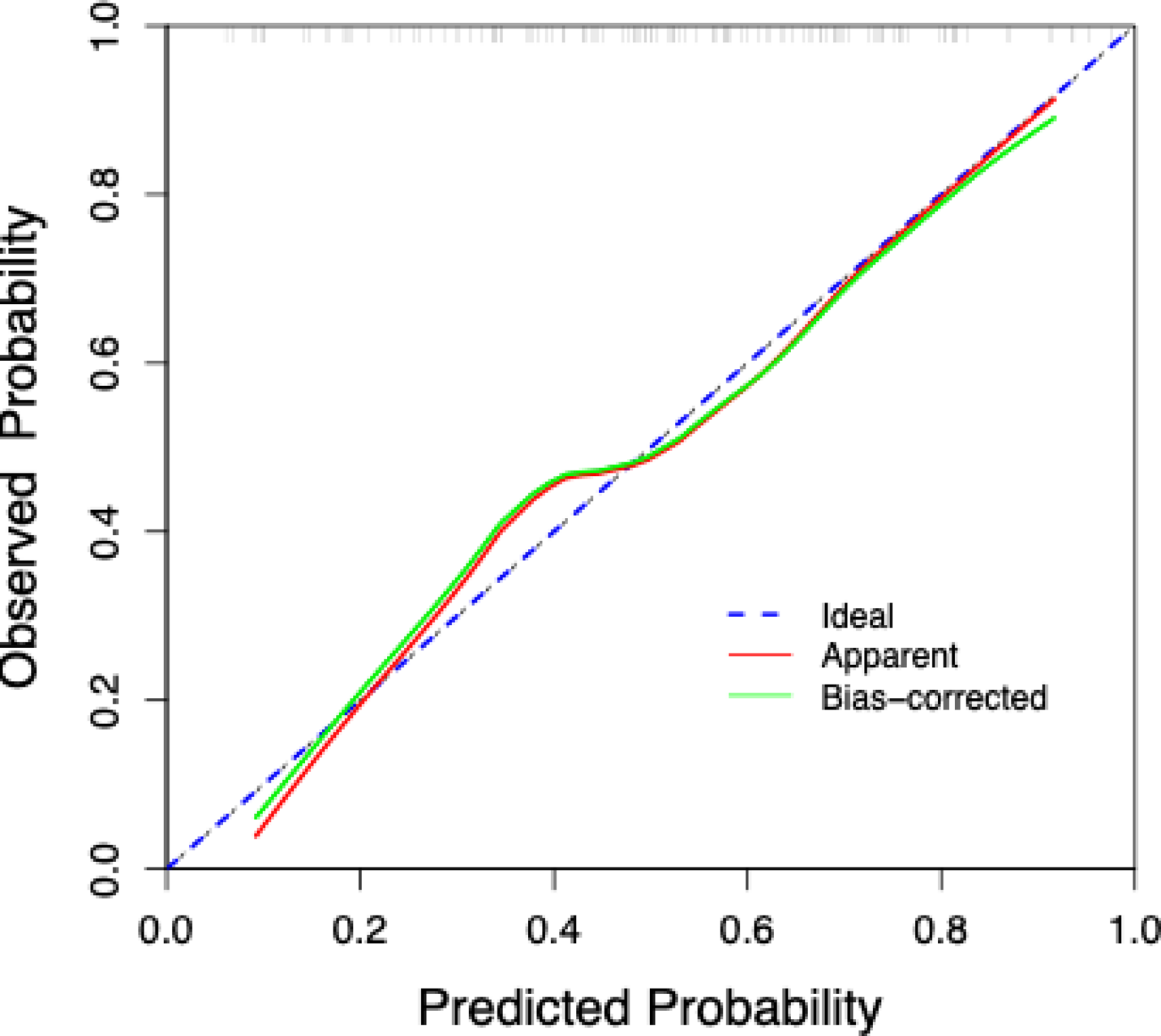
Calibration curves of the nomogram prediction. The x-axis and the y-axis represent the nomogram prediction and the actual situation, respectively. The diagonal line represents perfect agreement between predicted and observed probabilities.

**Figure 5:**
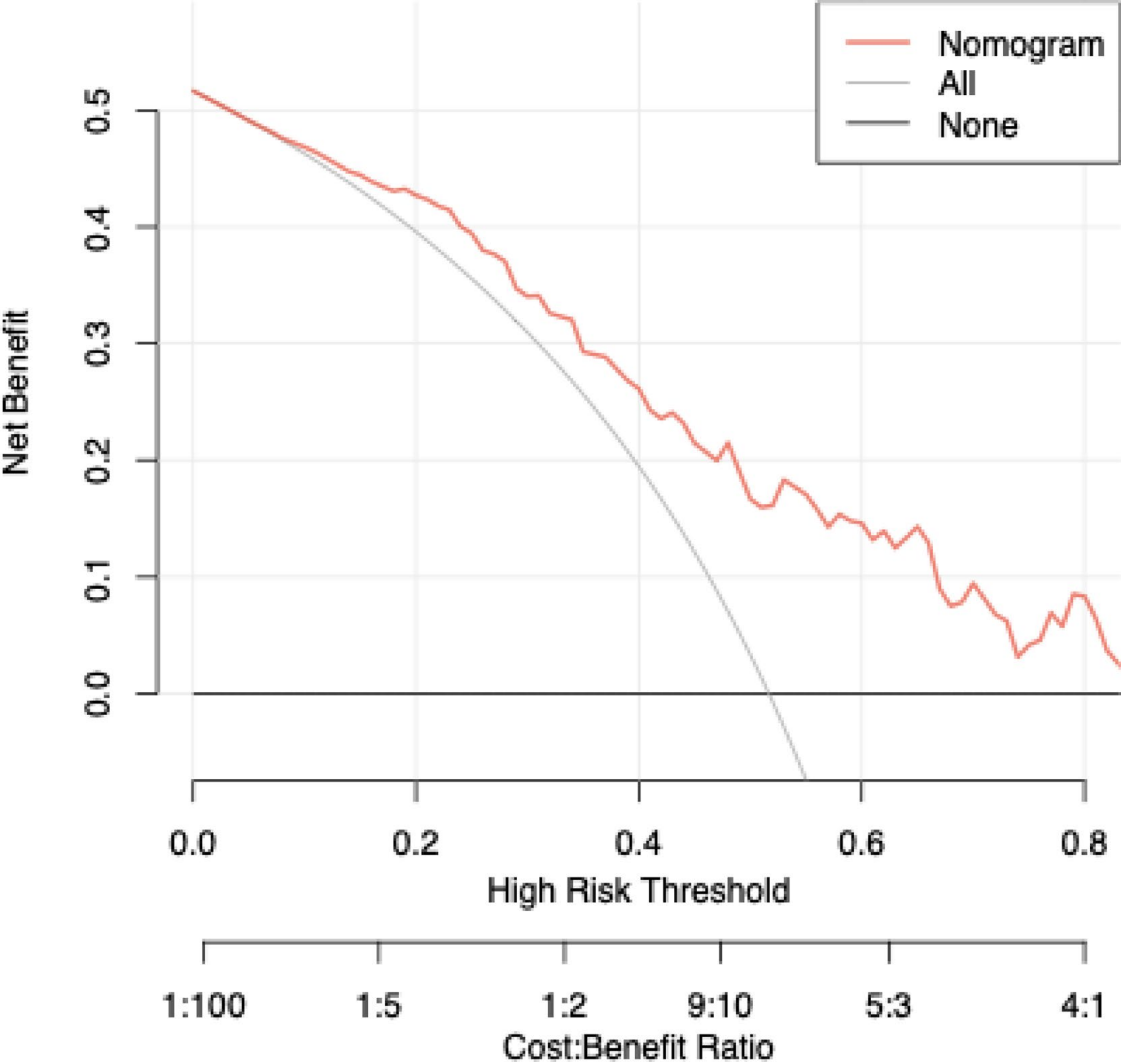
Decision curve analysis for the predictive model. The nomogram showed greater net benefit than the treat-all and treat-none strategies mainly across threshold probabilities of approximately 10%–60%; outside this range, incremental clinical value appeared limited.

Model performance was evaluated using tenfold stratified cross-validation, which yielded an average accuracy of 0.658 ± 0.129 and a mean AUC of 0.789 ± 0.151, indicating reasonable internal stability. The Firth’s penalized logistic regression model showed moderate discrimination, with an apparent AUC of 0.755 and an optimism-corrected AUC of 0.749 based on 1,000 bootstrap resamples (Fig. 3). The optimal probability cutoff determined by the Youden index was 0.552, with a sensitivity of 0.645, specificity of 0.759, positive predictive value of 0.741, negative predictive value of 0.667, and overall accuracy of 0.700 (Table 5). These values indicate that the nomogram should not be used as a stand-alone rule-in or rule-out tool for subsequent clinician-determined surgical intervention, but may provide an admission-stage risk signal when interpreted together with clinical assessment. Calibration was satisfactory within this cohort, as shown by a non-significant Hosmer–Lemeshow test (χ^2^ = 3.192, *P* = 0.922) and a Brier score of 0.198. The calibration intercept (− 0.002) and slope (1.025) were close to their ideal values, suggesting limited systematic overestimation or underestimation of risk (Fig. 4). The calibration plot showed close agreement between predicted and observed probabilities across most of the risk range, with minor deviation at the extremes. In decision curve analysis, the nomogram yielded greater net benefit than either the treat-all or treat-none strategies across threshold probabilities of approximately 0.10–0.60 (Fig. 5). Within this range, the model may support more informative risk stratification than default strategies alone; outside this range, its incremental value appeared limited. Overall, the model showed moderate discrimination and satisfactory within-cohort calibration for early risk stratification of subsequent clinician-determined surgical intervention. Because the outcome was partly shaped by clinical decision-making, these performance estimates should be interpreted with attention to potential inter-physician and inter-center variability.

## Discussion

We developed and internally validated an admission-based nomogram using AGR and CLR to estimate subsequent surgical intervention in children with AHO. Lower AGR and higher CLR at admission were independently associated with surgery during the index hospitalization. Compared with conventional decision-making, which often depends on serial clinical assessment and subsequent imaging, this model provides an earlier risk estimate based on routinely available biomarkers. The endpoint, however, was clinician-determined surgical intervention rather than an adjudicated measure of objective disease severity or biological necessity for surgery. This decision-dependent endpoint remains clinically relevant because it reflects real-world escalation to operative source control during the index hospitalization. The model was designed to identify, at admission, children who may be more likely to enter a surgical management pathway and who therefore warrant closer observation, earlier imaging prioritization, and timely reassessment by the pediatric orthopedic team. Admission AGR and CLR may serve as early composite markers of systemic inflammatory burden, nutritional-inflammatory status, and host immune response, thereby helping identify children who are more likely to enter a subsequent surgical management pathway before culture confirmation and detailed MRI characterization are available.

Previous studies have shown that laboratory parameters have prognostic value in pediatric acute hematogenous osteomyelitis, but most have focused on individual inflammatory markers rather than simple composite biomarkers available at admission. In the present study, several variables were associated with surgical intervention in univariable analyses, but only AGR and CLR were retained in the primary multivariable model. This parsimonious design should be understood in relation to the intended time point of model use. The exclusion of MRSA status, detailed MRI findings, abscess size, and concomitant septic arthritis does not mean that these factors are unimportant. They remain central to definitive surgical decision-making, especially abscess burden and septic arthritis. Incorporating them, however, would shift the model from an admission-stage warning tool to a later-stage operative decision model after microbiological and imaging information becomes available. The present model was deliberately designed to provide an early signal before culture confirmation and detailed MRI characterization, helping clinicians prioritize subsequent imaging, monitoring intensity, and reassessment rather than replacing later microbiological and imaging inputs. The expanded sensitivity analysis that included fever at admission, symptom duration, and neutrophil count supported the stability of AGR and CLR, but it does not establish that the two-biomarker model is sufficient for definitive risk classification.

The present findings can be placed alongside recent prediction work in pediatric osteomyelitis. Stephan et al. developed a 4-point clinical score that uses illness duration, fever, CRP, and ESR to guide diagnostic imaging in suspected AHO^20^. That model addresses the initial diagnostic phase, whereas our study estimates the likelihood of subsequent surgical intervention. Alhinai et al. developed A-SCORE and C-SCORE models to predict acute and chronic complications using data collected within the first 96 h of hospitalization^21^. By contrast, our model uses laboratory values obtained within two hours of presentation. Guo et al. specifically examined surgical intervention in pediatric AHO and reported a combined model with an AUC of approximately 0.76^12^, which is similar to the discrimination observed in the present nomogram. However, the two models differ in intended timing, predictor availability, outcome framing, and reproducibility. Our model uses only admission AGR and CLR, which are routinely available before culture confirmation and detailed MRI characterization, and reports the full regression equation to facilitate reproducibility. Although this simplicity should not be interpreted as readiness for immediate implementation, it supports the potential value of AGR and CLR as early, interpretable risk markers for admission-stage stratification. Importantly, when AHO is accompanied by suspected or confirmed septic arthritis, management decisions require disease-specific assessment of the joint infection and should integrate evolving clinical findings and imaging evaluation rather than rely solely on admission laboratory biomarkers^22^. Thus, the present nomogram should be interpreted as an early triage signal, not as a substitute for later microbiological, imaging, and source-control decisions. Future external validation should determine whether these markers can be incorporated into broader prediction frameworks together with microbiological, imaging, and clinical evolution variables.

AGR represents a valuable composite biomarker reflecting the patient’s nutritional status and systemic inflammatory response^23^. Lower AGR values indicate decreased serum albumin concentrations combined with elevated globulin levels, suggestive of intensified inflammation and possible nutritional compromise^24^. In our cohort, AGR values were significantly lower among surgical intervention cases compared with conservatively managed patients (1.4 vs. 1.6, *P* < 0.001), and multivariable Firth’s penalized logistic regression identified AGR as an independent protective factor against surgical intervention (OR 0.056, 95% CI 0.012–0.220, *P* < 0.001). The protective effect of higher AGR may relate mechanistically to albumin’s role as a negative acute-phase reactant^24^. Severe infections decrease hepatic albumin synthesis, enhance albumin leakage from capillaries, and exacerbate tissue edema, intramedullary pressure elevation, and potential abscess formation^25^. Concurrently, acute-phase globulins—including immunoglobulins and complement proteins—increase markedly during acute inflammation, further lowering AGR^24^. Indeed, previous studies consistently support AGR’s prognostic value in musculoskeletal infections. A meta-analysis by Chen et al. demonstrated that AGR reliably detects periprosthetic joint infection, achieving a pooled AUC of 0.88 (95% CI 0.85–0.91)^17^. Similarly, Shang et al. identified a low AGR as strongly associated with periprosthetic joint infection risk^16^. Furthermore, reduced AGR levels predict adverse outcomes in conditions beyond orthopedic infections, such as COVID-19 and various malignancies, underscoring the broader applicability of this marker in severe inflammatory conditions^26^. The consistent prognostic value of AGR across diverse inflammatory conditions suggests its fundamental role in systemic responses to infection and tissue injury.

CLR offers complementary insight into host immunity and inflammation dynamics. Elevated CLR values represent a state characterized by high inflammatory activity (elevated CRP) coupled with compromised immune response (lymphocytopenia)^27^. Our results indicated that CLR was significantly higher in surgically managed children compared with the conservative group (21.2 vs. 13.8; *P* = 0.019), and CLR independently predicted surgical intervention risk (OR 1.008, 95% CI 1.002–1.018; *P* = 0.008). Elevated CRP, an acute-phase protein, rapidly rises in response to inflammation, whereas lymphocyte counts typically decline in severe infections due to apoptosis or redistribution, reflecting immune suppression. Ullah et al. demonstrated that CLR upon admission reliably predicts severity and mortality outcomes in SARS-CoV-2 infections^28^. Similarly, critically ill sepsis patients with decreased lymphocyte-to-CRP ratios (inverse CLR) exhibited increased 30-day mortality and acute kidney injury rates^29^. Our findings extend this prognostic capability to pediatric AHO, suggesting elevated CLR reflects severe systemic inflammation and diminished immune resilience, which may prompt closer reassessment and source-control evaluation rather than directly determining surgical intervention.

Together, AGR and CLR capture complementary aspects of systemic inflammation, nutritional-inflammatory status, and host immune response. The discrimination of our nomogram was comparable to that of the combined model reported by Guo et al., with both models achieving an AUC of approximately 0.76^12^. An AUC of 0.755, together with a sensitivity of 0.645 and specificity of 0.759, indicates moderate discrimination and does not support use of the nomogram as a stand-alone operative decision tool. Its clinical value should instead be interpreted as an admission-stage risk signal based on routinely available laboratory markers, particularly before culture confirmation and detailed MRI characterization are available. False-low predictions could delay closer reassessment in children who later require operative management, whereas false-high predictions may lead to additional evaluation or closer observation in children who may not ultimately require operative management. The decision curve suggested potential net benefit mainly across threshold probabilities of approximately 10%–60%, representing a zone of clinical uncertainty in which model-informed risk stratification may help prioritize observation intensity, MRI evaluation, and pediatric orthopedic reassessment. Outside this range, the model’s incremental value appears limited. In practice, a higher predicted probability should prompt earlier reassessment and diagnostic prioritization rather than immediate operative intervention. Operative management must continue to integrate symptoms, serial inflammatory trends, microbiological findings, MRI features, abscess burden, septic arthritis, and local expertise.

This study has several limitations. First, its retrospective, two-center design may introduce selection bias, information bias, and unmeasured heterogeneity in documentation, imaging timing, and clinical management. Although both centers followed broadly similar treatment principles, operative indications were not prospectively standardized, and inter-physician or inter-center variation may have affected both the outcome definition and model performance. Second, the primary outcome was surgical intervention during the index hospitalization, a decision-dependent endpoint shaped by clinical progression, imaging findings, microbiological information, and physician judgment. Therefore, the model estimates clinician-determined surgical management, a pathway that incorporates disease severity but is not equivalent to an independently adjudicated measure of objective disease severity or biological necessity for surgery, which limits external interpretability. Third, the model underwent internal validation only. Bootstrap and cross-validation procedures assess optimism and internal stability but do not establish transportability to hospitals with different MRI access, antimicrobial practices, MRSA prevalence, or surgical thresholds. External validation in independent, preferably multicenter cohorts is required before clinical implementation. Fourth, the model’s moderate discrimination and limited decision-curve threshold range indicate that it should serve as an adjunct to routine assessment, particularly for admission-stage triage and reassessment prioritization; it should not be used as the sole basis for excluding subsequent surgical management or mandating intervention. Fifth, the parsimonious design excluded important variables such as MRSA status, detailed MRI features, abscess size, and concomitant septic arthritis. These variables are clinically important but were not uniformly available at the intended admission-stage point of use, and their omission may have introduced residual confounding. Finally, although missingness was low and complete-case analysis yielded materially similar results, median imputation may underestimate uncertainty. Future prospective studies should standardize surgical criteria, laboratory measurement procedures, imaging definitions, and missing-data handling while externally validating and updating the model.

## Conclusion

This two-center retrospective study suggests that admission AGR and CLR may provide an early, readily available signal associated with subsequent clinician-determined surgical intervention in pediatric AHO. The nomogram may support admission-stage risk stratification and earlier reassessment, but should be considered exploratory and hypothesis-generating until externally validated.

## Data Availability

The original contributions presented in the study are included in the article, further inquiries can be directed to the corresponding author.

## Abbreviations

AHO: Acute hematogenous osteomyelitis
MRSA: Methicillin-resistant Staphylococcus aureus
MSSA: Methicillin-susceptible Staphylococcus aureus
MRI: Magnetic resonance imaging
CRP: C-reactive protein
ESR: Erythrocyte sedimentation rate
WBC: White blood cell count
CLR: C-reactive protein to lymphocyte ratio
AGR: Albumin to globulin ratio
ROC: Receiver operating characteristic
AUC: Area under the curve
PPV: Positive predictive value
NPV: Negative predictive value
